# Association of network connectivity via resting state functional MRI with consciousness, mortality, and outcomes in neonatal acute brain injury

**DOI:** 10.1016/j.nicl.2022.102962

**Published:** 2022-02-09

**Authors:** Varina L. Boerwinkle, Bethany L. Sussman, Iliana Manjón, Lucia Mirea, Saher Suleman, Sarah N. Wyckoff, Alexandra Bonnell, Andrew Orgill, Deborah J. Tom

**Affiliations:** a Division of Pediatric Neurology, Barrow Neurological Institute at Phoenix Children’s Hospital, 1919 E. Thomas Rd, Phoenix, AZ 85016, USA; b Department of Neuroscience Research, Barrow Neurological Institute at Phoenix Children’s Hospital, 1919 E. Thomas Rd, Phoenix, AZ 85016, USA; c Department of Clinical Research, Phoenix Children’s Hospital, 1919 E. Thomas Rd, Phoenix, AZ 85016, USA; d Division of Neonatology, Phoenix Children’s Hospital, 1919 E. Thomas Rd, Phoenix, AZ 85016, USA; e University of Arizona College of Medicine – Tucson, 1501 N. Campbell Ave, Tucson, AZ 85724, USA

**Keywords:** ABI, acute brain injury, DoC, disorders of consciousness, EEG, electroencephalogram, fMRI, functional magnetic resonance imaging, RSN, resting state network(s), DMN, default mode network, FP, frontoparietal network, Lang/FP, language/frontoparietal network, HIE, hypoxic ischemic encephalopathy, a-MRI, anatomical MRI, RS, resting state functional magnetic resonance imaging, MRS, magnetic resonance spectroscopy, cv-EEG, continuous video EEG, ICA, independent component analysis, BOLD, blood oxygenation level dependent signal, DRE, drug resistant epilepsy, FE, Fisher exact, odds ratio, OR, CI, confidence intervals, OLR, ordinal logistic regression analyses, MLR multinomial logistic regression models, CC, correlation coefficient, TBI, traumatic brain injury, Resting state functional MRI (rs-fMRI), Neonatal acute brain injury (ABI), Hypoxic ischemic encephalopathy (HIE), Connectivity, Consciousness

## Abstract

**Background:**

An accurate and comprehensive test of integrated brain network function is needed for neonates during the acute brain injury period to inform on morbidity. This retrospective cohort study assessed whether integrated brain network function acquired by resting state functional MRI during the acute period in neonates with brain injury, is associated with acute exam, neonatal mortality, and 6-month outcomes.

**Methods:**

Study subjects included 40 consecutive neonates with resting state functional MRI acquired within 31 days after suspected brain insult from March 2018 to July 2019 at Phoenix Children’s Hospital. Acute-period exam and test results were assigned ordinal scores based on severity as documented by respective treating specialists. Analyses (Fisher exact, Wilcoxon-rank sum test, ordinal/multinomial logistic regression) examined association of resting state networks with demographics, presentation, neurological exam, electroencephalogram, anatomical MRI, magnetic resonance spectroscopy, passive task functional MRI, and outcomes of discharge condition, outpatient development, motor tone, seizure, and mortality.

**Results:**

Subjects had a mean (standard deviation) gestational age of 37.8 (2.6) weeks, a majority were male (63%), with a diagnosis of hypoxic ischemic encephalopathy (68%). Findings at birth included mild distress (48%), moderately abnormal neurological exam (33%), and consciousness characterized as awake but irritable (40%). Significant associations after multiple testing corrections were detected for resting state networks: basal ganglia with outpatient developmental delay (odds ratio [OR], 14.5; 99.4% confidence interval [CI], 2.00–105; *P* < .001) and motor tone/weakness (OR, 9.98; 99.4% CI, 1.72–57.9; *P* < .001); language/frontoparietal network with discharge condition (OR, 5.13; 99.4% CI, 1.22–21.5; *P* = .002) and outpatient developmental delay (OR, 4.77; 99.4% CI, 1.21–18.7; *P=*.002); default mode network with discharge condition (OR, 3.72; 99.4% CI, 1.01–13.78; *P=*.006) and neurological exam (*P = .*002 (FE); OR, 11.8; 99.4% CI, 0.73–191; *P = .*01 (OLR)); and seizure onset zone with motor tone/weakness (OR, 3.31; 99.4% CI, 1.08–10.1; *P=*.003). Resting state networks were not detected in three neonates, who died prior to discharge.

**Conclusions:**

This study provides level 3 evidence (OCEBM Levels of Evidence Working Group) demonstrating that in neonatal acute brain injury, the degree of abnormality of resting state networks is associated with acute exam and outcomes. Total lack of brain network detection was only found in patients who did not survive.

## Highlights

• Basal ganglia and seizure onset zone networks were associated with motor outcomes.
• Broad language/cognitive region networks were associated with developmental delay.
• Discharge with mortality was linked to default mode and language/cognitive networks.
• Exams were not linked to networks after multiple testing corrections.
• Lack of detection of all studied networks only occurred in those who did not survive.

## Graphical abstract

**Figure:**
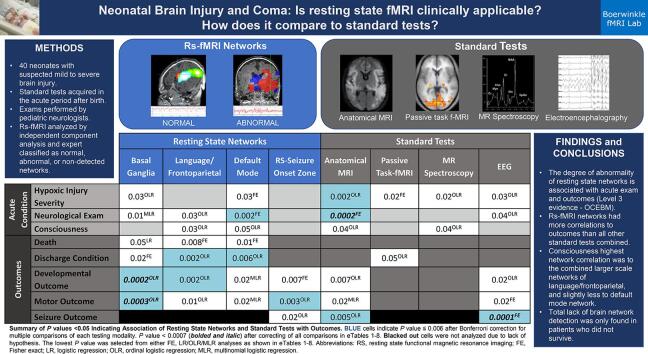

## 1 Introduction

In neonatal acute brain injury (ABI), exam and test markers of integrated brain network function are needed to characterize disorders of consciousness (DoC) and recovery potential, which are used to inform withdrawal of life sustaining therapy determinations with profound consequences (Demertzi et al., 2011, Douglas-Escobar and Weiss, 2015, Downar et al., 2016, Kondziella et al., 2020, Michel et al., 2019, Pant, 2021, Turgeon et al., 2011). DoC misdiagnosis is estimated in 40% and 70% of adults (Schnakers et al., 2009), due to confounds including complex movements which may be intentional, seizures, reflexes or automatisms (Han et al., 2006, Saposnik et al., 2005). In neonates, DoC diagnosis may be higher or misdiagnosed due to lack of DoC definitions, validated exam markers, and diagnostics (Bruno et al., 2011, Giacino et al., 2018, Machado et al., 2008, Nakagawa et al., 2012, Schnakers et al., 2009), which likely reduce precision in diagnosis-informed treatments, outcome predictions, and well-informed withdrawal of life sustaining therapy determinations (Devictor et al., 2008, Downar et al., 2016, Pant, 2021, Turgeon et al., 2011).

Integrated brain network function is pivotal in adult DoC diagnosis and recovery prediction (Falletta Caravasso et al., 2016, Giacino et al., 2018, Hahn et al., 2021, Kondziella et al., 2017, Michel et al., 2019, Rosazza et al., 2016, Sair et al., 2018, Silva et al., 2015). Integrated brain network function modalities include stimulation/task-based and resting state electroencephalogram (EEG) and functional magnetic resonance imaging (fMRI). These advanced measures supplant the exam when the former indicate higher capacity for consciousness in adults with subacute to chronic DoC (Kondziella et al., 2020). However, the need for such diagnostic precision is greater in acute than chronic brain injury given the higher mortality and rate of withdrawal of life sustaining therapy in the acute period. This likely also effects pediatric and neonatal patients.

Among the integrated brain network function modalities available for DoC, much progress has been made in neonates with resting state functional MRI (RS). In *healthy* neonates, resting state networks (RSNs) are detectable by the 3rd trimester (Doria et al., 2010). The default mode (DMN), attention, and frontoparietal (FP) networks are detectable by term (Fransson et al., 2009, van den Heuvel et al., 2015). In neonates with *mild to mild-moderate* ABI, RSN metrics are predictive of neurodevelopmental outcomes (Damoiseaux et al., 2006, Doria et al., 2010, Ferradal et al., 2018, He et al., 2018, Jenkinson et al., 2002, Mak et al., 2018, Shi et al., 2018, Smith et al., 2009). Prematurity affects long-range and frontal connectivity, (Bouyssi-Kobar et al., 2019, Della Rosa et al., 2021, He et al., 2018, Linke et al., 2018, Rogers et al., 2017, Smyser et al., 2010, Strahle et al., 2019, Wheelock et al., 2018) and hypoxic ischemic encephalopathy (HIE) (Li et al., 2019) affects the language, vision, and sensory-motor networks. In *mild to moderate* ABI, RS is also predictive of neurodevelopmental outcomes (Bouyssi-Kobar et al., 2019, Della Rosa et al., 2021, He et al., 2018, Li et al., 2019, Linke et al., 2018, Rogers et al., 2017, Smyser et al., 2010, Strahle et al., 2019, Wheelock et al., 2018).

However, only one large neonatal-ABI-RS study included ***severe*** ABI: the population most likely to have withdrawal of life sustaining therapy determinations in the acute period, anatomical MRI (a-MRI), exam, and brain-behavior evaluation. This study was performed on a 1.5 Tesla MRI, which has significantly lower fMRI signal detection capacity than higher Tesla MRI (Krasnow et al., 2003). The study showed RS-motor outcome association, ***irrespective of pathological grouping***, with demographics, presentation, course-severity clinical factors, and a-MRI severity, while relationships between RSN, mortality, acute exam, and a-MRI were not determined (Linke et al., 2018, Ní Bhroin et al., 2021). Lastly, RSN alterations from perinatal brain injury were detectable years later and correspond to developmental outcomes, indicating the RS findings from neonatal ABI are durable and meaningful (Ní Bhroin et al., 2021). Thus, the gap in utility of RS in the full spectrum of ABI severity against outcomes beyond motor is largely unknown, and validation of the original motor finding is needed.

Importantly, developing biomarkers of network pathology in heterogeneous cohorts of brain injury is of high priority according to recent expert opinion consensus (Edlow et al., 2020, Kondziella et al., 2020, LaRovere and Tasker, 2020, Provencio et al., 2020). The expert consensus reasoning is that the effect on integrated brain network function of the brain insult, rather than the insult subtype, is thought to be the most likely determining factor in capacity for recovery. Thus, a narrowed injury-causation-subtype-designed trial is thought to be less favorable in demonstrating ultimate clinical utility.

Presently, study of the relationship between the integrated brain network function and the controversial consciousness exam in neonates is needed. Without supratentorial network activity confirmation, such as EEG sleep-wake brain-state changes, neonatal consciousness behaviors have been proposed by some to be brainstem-driven, with no link to recovery potential (Ashwal et al., 1990). Alternatively, non-anencephalic, stimulus-brainstem-driven neonatal behaviors may still predict future consciousness (del Giudice et al., 2016, Ishaque et al., 2017). Supportively, lack of arousal behavior is reliable enough, by current expert consensus, to be a key component of the brain death determination in neonates (Nakagawa et al., 2011, Nakagawa et al., 2012).

This study aims to evaluate RS in *mild to severe* neonatal ABI with acute-period neurological/consciousness exam, mortality, tests, and outcomes.

## 2 Methods

Study patients included consecutive ABI neonates with clinical RS and MRI evaluated as standard of care during March 2018 to July 2019 at Phoenix Children’s Hospital. The institutional review board approved this retrospective study with waived consent.

Data were collected from the medical records including demographics (sex, gestational age), diagnosis, presentation, exams/tests (neurological exam, consciousness, a-MRI, task-fMRI, RS, magnetic resonance spectroscopy (MRS), EEG) during the acute period, discharge condition, mortality, and outpatient outcomes (development, motor tone, strength, seizure).

The acute-period and diagnosis, exam, acute tests findings, and outcomes were categorized using ordinal scores based on factors documented by the respective treating pediatric specialists, as further described in the following sections 2.1 through 2.5. If the element’s categorization exact wording was not specifically documented within the treating physician’s acute-period charted note, then two separate, blinded study personnel made the determination based on the wording available. A third research personnel was available if disagreement occurred, however, none occurred. The acute-period HIE severity, diagnoses, presentation, and neurological and consciousness exams were documented prior to the MRI with RS scans. Initial EEG reads occurred after HIE and presentation severity determination, but prior to the MRI scan and final diagnosis determinations.

The number of patients receiving each assessment is shown in Table 1. Some patients did not have all tests done (i.e. task-fMRI, EEG, MRS) because they were not clinically indicated. No additional tests were done for the purpose of this study. Subjects with missing data due to tests not performed were excluded from analyses of respective tests.

**Table 1:** Demographics and Ordinal Scores of Acute Clinical Findings Tests and Outcomes.

	**Factor**	**Ordinal Score**	**N = 40**
**Age (weeks), Sex**	Mean Age (SD)		37.8 (2.6)
	Male, N (%)		25 (63)
	Mean Age at RS ^e^ (SD)		
	HIE		1.6 (2.0)
	Non-HIE		3.1 (3.1)
	Median Outpatient Age at FU (Q1, Q3)		31.6(13.9, 37.6)
**Diagnosis**	**HIE Severity (Sarnat criteria),** N (% of 27)	1: Mild	14 (52)
		2: Moderate	7 (26)
		3: Severe	6 (22)
	**Non-HIE**, N (% of 13)	Genetic diagnosis or congenital malformation	4 (31)
		Neonatal epilepsy	3 (23)
		Traumatic brain injury	2 (15)
		Infection	1 (8)
		Hypoglycemia	1 (8)
		Arterial or venous ischemic infarction	2 (15)
**Acute Condition**	**Presentation**, N (%) ^a^	0: Normal without distress	1 (2)
		1: Mild distress requiring airway support <48 h	19 (48)
		2: Moderate distress requiring airway support >48 h or cardiovascular intervention	9 (22)
		3: Severe distress with cardiovascular intervention and/or ventilatory support >5 days)	11 (28)
	**Neurologic Exam**, N (%) ^b^	0: Normal	10 (25)
		1: Mildly abnormal (single extremity weakness or mild tone differences; <48 h of any atypical movement)	12 (30)
		2: Moderately abnormal (≥2 weak extremities, hypotonic, moderately increased tone, 48 h to 5 days of any atypical movements)	13 (33)
		3: Severely abnormal (flaccid, marked spasticity or dystonia, >5 days of atypical movements)	5 (12)
	**Consciousness (Day 0**–**5)**, N (%) ^b^	0: Normal (open eyes and moves normally in response to nonpainful environmental stimuli including light, light touch, or soft voice)	9 (22)
		1: Irritable or sleeping more than typical but arouses easily and moves to touch normally)	16 (40)
		2: Wakes up only to painful stimulation (and less spontaneous movements than typical)	11 (28)
		3: Coma (does not open eyes, no movement after environmental stimuli, painful stimulation, or only reflexive-appearing abnormal movements)	3 (8)
		4: Episodically arousable (alternating normal to unresponsive, suggestive of seizure)	1 (2)
**Acute Tests**	**Anatomical MRI**, N (%) ^c^	0: Normal	15 (38)
		1: Mildly abnormal	11 (28)
		2: Moderately abnormal	5 (12)
		3: Severely abnormal	9 (22)
	**Task-fMRI**, N (%) ^c^	0: Normal	30 (75)
		1: Mildly abnormal	0 (0)
		2: Moderately abnormal	3 (8)
		3: Severely abnormal	3 (8)
		4: Not done	4 (10)
	**MRS**, N (%) ^c^	0: Normal	12 (30)
		1: Mildly abnormal	5 (12)
		2: Moderately abnormal	1 (2)
		3: Severely abnormal	3 (8)
		4: Not done	19 (48)
	**EEG**, N (%) ^d^	0: Normal	3 (8)
		1: Mild (not flat) background abnormality only	22 (55)
		2: Seizure	13 (33)
		3: Flat amplitude	1 (2)
		4: Not done	1 (2)
**RSN**	**BG**, N (%)	0: Normal	10 (25)
		1: Detected with atypical features	24 (60)
		2: Not detected	6 (15)
	**Lang/FP**, N (%)	0: Normal	23 (58)
		1: Detected with atypical features	13 (32)
		2: Not detected	4 (10)
	**DMN**, N (%)	0: Normal	27 (68)
		1: Detected with atypical features	8 (20)
		2: Not detected	5 (12)
	**RS-SOZ**, N (%)	0: Normal	24 (60)
		1: Low or indeterminate consistency with RS-SOZ	7 (18)
		2: Highly consistent with RS-SOZ	9 (22)
**Outcomes**	**Discharge Condition**, N (%) ^a^	0: Normal	19 (48)
		1: Mild support such as gavage or supplemental O_2_	16 (40)
		2: Moderate deficits	2 (5)
		3: Decease	3 (7)
	**Outpatient Developmental Delay**, N (% of 36) ^b^	0: Normal	19 (49)
		1: Mild delay	2 (5)
		2: Moderate delay or focal finding on exam	10 (26)
		3: Severe findings on exam (requiring gastrostomy tube feeds, or hospitalization-style care)	5 (13)
		4: Deceased	3 (8)
	**Outpatient Motor-tone**, N (%) ^b^	0: Normal	18 (46)
		1: Mildly increased tone or weakness	3 (8)
		2: Moderately increased tone or weakness	10 (26)
		3: Severely increased tone or weakness	5 (13)
		4: Deceased	3 (8)
	**Seizure,** N (% of 36) ^b^	0: No	23 (64)
		1: Yes (EEG, neurologist exam, or witnessed seizure; or neurologist determination of need for continued antiseizure medication)	13 (36)
	**Repeat Insult**, N (%) ^b^	0: No known insult	36 (90)
		1: Yes (after RS)	1 (2)
		2: Not applicable	3 (8)
	**Death**, N (%) ^b, c^	Yes	3 (8)

Categorization as documented by ^a^ neonatologist, ^b^ neurologist, ^c^ neuroradiologist, ^d^ epileptologist. ^e^ The exception to RS timing was one patient who had RS at 70 days of age due to complex and poor course followed by late transfer and evaluation at our institution. Abbreviations: RS, resting state functional magnetic resonance imaging; SD, standard deviation; HIE, hypoxic ischemic encephalopathy; FU, follow-up; EEG, electroencephalogram; MRI, magnetic resonance imaging, MRS, magnetic resonance spectroscopy; RSN, resting state network(s); BG, basal ganglia network; Lang/FP, language and/or frontoparietal network; DMN, default mode network; RS-SOZ, resting-state seizure onset zone or abnormal findings concerning for seizure related pathology.

### 2.1 Acute-period diagnoses

HIE was determined by Sarnat-criteria (Mrelashvili et al., 2020), as documented by the neonatologist. HIE severity and non-HIE diagnoses were determined by the discharging neonatologist’s final report.

Presentation severity/distress categorization was provided by the treating neonatologist’s determination for the need of airway or ventilatory support and cardiovascular intervention, and the duration of respective interventions, as detailed in Table 1.

### 2.2 Neurological and consciousness exams

Neurological and consciousness exams were performed by the initial consulting neurocritical care provider (pediatric neurologists) typically on the day of admission (or the following day if admitted overnight). Repeat exams were documented if new additional events occurred, including new weakness, worsened encephalopathy, movements concerning for seizure, or new abnormal findings on the EEG. Validated scales are lacking for neonatal acute-period factors and outcomes that are validated in a mixed pathology and full-spectrum severity, the *peri*-term gestation population, or any neonatal population related to consciousness. Thus, these elements were categorized by ordinal scales designed to capture the severity range based on the neurologist’s documentation of weakness, tone difference, abnormal movements, lack of normal movement, and length of time of ongoing difference from normal, as delineated in Table 1.

Repeat insult: To differentiate outcomes related to the acute-period exams, differentiation between those with a second separate brain insult were recorded. Those who died prior to discharge were separately noted.

Death: Only those who died prior to discharge were recorded. No further deaths occurred at time of last available study follow-up.

### 2.3 Outcome diagnoses and exams

Discharge condition was determined by the discharging neonatologist according to their documentation of ongoing need for gavage feeding or supplemental oxygen being the mild category, and all other ongoing support or detectable abnormalities being categorized as “moderate deficit”. The most severe category was mortality.

Outpatient development, motor-tone, and seizure were determined by the pediatric neurologist through the last available follow-up (within 6 months) by authorized study collection (Table 1). Developmental delays diagnosed in the medical record and any ongoing focal neurological deficits factored into the follow-up “outpatient development” categorization. Severe findings on exam included ongoing need for gastrostomy tube feeds, airway clearance by deep suctioning, or ventilatory support. The “motor-tone” outcome is an abbreviated combination of motor abnormalities including both weakness and tone. The severity rating was based entirely on the documenting neurologist’s impression of degree of difference from normal (Table 1). The “seizure” outcome denotes an ongoing concern for either a possible seizure occurring after discharge, either by EEG-capture, neurologist’s exam, caretaker witnessed event, *or* treating neurologist’s impression of need for ongoing anti-seizure medication.

### 2.4 Acute-period tests

#### 2.4.1 ICU MRI safety, anesthesia, and scan timing

To receive a clinical MRI, all patients needed to be safe and stable for transport to the scanner by the neonatologist and received continuous observation from the respective hospital care teams. Total scan time was approximately one hour. No safety event occurred during or related to scanning. As this is a retrospective study, all evaluations followed standard of care practices without any modifications.

At the time of scan, patients were not receiving anesthetic agents, as per their care plans. To minimize movement during imaging, infants were fed and swaddled, with multiple levels of ear protection, including earplugs, earmuffs, and standard headphones. Awake infants were rescheduled for later scan times as needed. Neonates with HIE, as diagnosed by their treating neonatologist (Azzopardi et al., 2009, Jacobs et al., 2011), received 48 h of therapeutic hypothermia and medications for symptomatic relief of cold temperature. After the end of cooling, clinical MRI with RS was performed. Infants with non-HIE brain MRI indications, received MRI with RS at more variable times according to clinical indications within the first month of life (Table 1) except for 3 neonates that had theirs done 59–73 days after birth for prognostication or presurgical evaluation. Most RS were preformed within the first 14 days of life.

Image and EEG severity categorization were determined by the interpreting pediatric neuroradiology (a-MRI, diffusion-weighted imaging (DWI), MRS, and task-fMRI), pediatric neurologist (with expertise in over 2000 individual age 0–21-year-old’s clinical RS interpretations), and pediatric epileptologist (reading EEG).

EEG was performed throughout the acute period. A standard array of 19 leads with continuous video EEG (cv-EEG) was interpretated via visual inspection by the pediatric epileptologist. Seizures on traditional EEG were defined as rhythmic discharges evolving in frequency, amplitude, morphology, and/or spatial distribution, lasting at least 10 s. Infants with suspected HIE received cv-EEG starting on the first day of admission and was removed on the day of MRI.

#### 2.4.2 a-MRI and MRS

All MRI sequences were acquired on a 3 Tesla MR scanner (Ingenia, Philips Healthcare, Best, The Netherlands) with a 32-channel headcoil. T1, T2, and diffusion-weighted sequences with corresponding apparent diffusion maps were acquired. MRS data were acquired using a single-voxel point resolved spectroscopy sequence localized to the white matter of the centrum semiovale, and grey matter of the basal ganglia region. Interpretation of a-MRI, DWI, and MRS was performed by the pediatric neuroradiologist.

#### 2.4.3 Task-fMRI

Subjects who remained still also received a 5-minute passive task-MRI. Passive movement of the right index finger by the MRI technician was performed. A positive test result was statistically significant motor cortex activation (Seghier et al., 2006). A visual paradigm of a kaleidoscope pattern projection was alternated with fixed cross visual presentation. A positive test was statistically significant activation within the occipital cortices. Images were processed using DynaSuite Neuro Software (Invivo Corporation, Gainesville, FL), which involves motion correction, co-registration of the fMRI data set with T1-weighted MRI images and generalized linear model processing of block paradigm-type designs. The task-fMRI was visually inspected for expected specific recorded real-time network activation. Resultant SensaVue general linear model difference between activation and baseline images were interpreted by the pediatric neuroradiologist.

#### 2.4.4 Rs

T2-weighted images were acquired with a repetition time (TR) 2000 ms, echo time (TE) 30 ms, matrix size of 80 × 80, flip angle of 80 degrees, number of slices to cover at least the supratentorium, slice thickness such that voxel size is 3.4 × 3.4 × 3.4 mm^3^, no slice gap, and inter-leaved top-down acquisition. The number of total volumes was 600, split between two equally timed runs, each approximately 10 min. Standard RS preprocessing steps were applied: non-brain structures were removed, the first 5 volumes were deleted to remove T1 saturation effects, high pass filter 100 s, inter-leaved slice time correction, minimal spatial smoothing at 1 mm, and motion corrected by MCFLIRT (Boerwinkle et al., 2017, Jenkinson et al., 2002). All subjects had less than 0.5 mm motion-induced displacement in any direction. Individual functional scans were registered to the patient’s high-resolution anatomical scan by using linear registration (Jenkinson et al., 2002, Jenkinson and Smith, 2001), with optimization using boundary-based registration (Greve and Fischl, 2009).

**Fig. 1:**
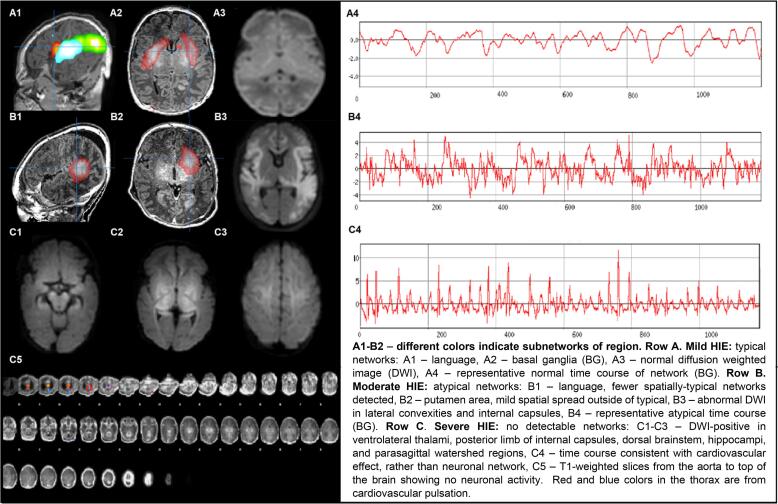
Resting-State Networks (RSN) and Diffusion Weighted Images (DWI) in Neonates with Mild to Severe Hypoxic Ischemic Encephalopathy (HIE).

RS independent component analysis (ICA), a data driven approach, was performed as in prior work (Beckmann and Smith, 2004, Boerwinkle et al., 2017, Griffanti et al., 2017, Mongerson et al., 2017). The FMRIB Software Library tool MELODIC analysis software was used for this study. The total number of detected independent components (IC) per patient was determined using automated dimensionality estimates based on established Bayesian computation, and an IC threshold of *P* < .05 was established by the standard local false discovery rate for IC detection (Beckmann and Smith, 2004, Boerwinkle et al., 2017). The results were manually sorted into noise versus neuronal signal. Expert visual interpretation of RS ICA outcomes in patients age 0–21 years with mixed causative pathologies including brain injury have been reported (Boerwinkle et al., 2019a, Boerwinkle et al., 2018a, Boerwinkle et al., 2018b, Boerwinkle et al., 2020, Boerwinkle et al., 2017, Boerwinkle et al., 2019b, Desai et al., 2018). Noise components, for example, were located outside of the brain in the cerebral spinal spaces and over major arteries, whereas known RSNs were located primarily over the grey matter with well published spatial patterns (Boerwinkle et al., 2017). Further information regarding RS acquisition, ICA, and the RS independent component classification approach can be found in the supplemental material (visually represented in **Figure 1** **of accompanied Method X article**). Normal RSNs were those detected with spatial consistency as previously described (e.g. motor, vision, default mode) and with blood oxygenation level dependent signal (BOLD) frequency <10 Hz/100 (defined as normal for neonate herein) (Boerwinkle et al., 2019a, Boerwinkle et al., 2017). This was supported by prior work wherein visual inspection of spatial consistency was compared to co-registered volumetric adult comparisons (Linke et al., 2018).

### 2.5 Resting state networks and seizure onset zones

Although RSNs are detectable in the neonatal population, known maturational changes still occur after this period, especially through increasing long-range connectivity. In the neonate’s RS clinical reports, each non-noise RSN is shown with its corresponding BOLD signal time course and associated frequency power spectrum. Examples of these RSNs and BOLD signals are found in Fig. 1 below. However, due to the need for a data reduction step for the study, the RSNs evaluated for study evaluation were: (1) the basal ganglia (BG), due its relationship with frequency of insult in neonates leading to motor disorders (Ní Bhroin et al., 2021); (2) the language/frontoparietal network (Lang/FP), due to the relationship of these networks with cognitive and communication capacities (Li et al., 2019); (3) the default mode network (DMN), due to its relationship with consciousness, and outcomes after brain injury (Linke et al., 2018, Ní Bhroin et al., 2021); and (4) atypical (non-RSN) networks, which were primarily located over grey matter, and did not have features consistent with published RSNs or RS noise (Boerwinkle et al., 2019a, Boerwinkle et al., 2017). The language/frontoparietal networks were combined to reduce the total number of comparisons, since nuanced differences in language and cognitive outcomes were not evaluated specifically.

Atypical networks were further categorized as either consistent with RS-SOZ, low or indeterminate consistency with RS-SOZ, or not consistent with RS-SOZ, according to the spatial and temporal features developed in children with drug resistant epilepsy (DRE) (Boerwinkle et al., 2019a, Boerwinkle et al., 2017). Of note, the timing of actual clinical or electrographic seizures after brain injury is highest in the immediate period (1–3 days), whereas the RS was typically performed by day 4 or after. Also, actively seizing neonates are not transferred for elective MRI. Thus, ongoing clinical seizures did not occur during the scan and seizures electrographic seizures were not measured during or near the time of the scan. Those neonates who did have seizures were continued on anti-seizure medication during the time of scan. Similarly, in children with DRE, findings by RS seemingly consistent with a SOZ are acquired almost entirely during the interictal period and are, thus, thought to indicate ongoing abnormal network activity that occurs at sub-clinical seizure threshold. Ultimately, these findings have shown to be reliable to direct neurosurgical intervention leading to improved surgical outcomes (Boerwinkle et al., 2019a, Boerwinkle et al., 2017, Chakraborty et al., 2020). However, being that this population does not have the diagnosis of DRE, the meaning of RS-SOZ after ABI in those with largely resolved seizures may have more to do with the neuroplastic changes of the injury, rather than correlation with a resolved seizure or prediction of epileptogenicity.

Lastly, those subjects missing one of these RSNs or with no RSNs detected were noted because the poorest RS connectivity is reported in the few cases after brain death (Boly et al., 2009, Han et al., 2006, Jain and DeGeorgia, 2005, James, 1998, Jørgensen and Malchow-Møller, 1981, Kumar et al., 2016, Marti-Fabregas et al., 2000, Saposnik et al., 2005). Thus, the investigators expected lack of RSN detection to occur more often in those with poorer outcomes, though that would likely be determined only on a descriptive level (though still interesting for informing future prospective trial design) as survival after ABI in neonates is fairly robust.

### 2.6 Statistical analyses

The distribution of demographics, clinical factors, and outcomes were summarized using descriptive statistics, and compared between RSN categories using the Fisher exact (FE) or Kruskal-Wallis test, as appropriate for the data distribution. The magnitude of association between RSN category scores and factors/outcomes was quantified using odds ratio (OR) estimates and corresponding confidence intervals (CI) from ordinal logistic regression analyses (OLR), or multinomial logistic regression models (MLR) when the proportional odds assumption was not valid. Similar analyses examined association of clinical factors and outcomes with findings from EEG, a-MRI, task-fMRI, and MRS modalities. For OLR/MLR models, the modality predictor ordinal levels were fit as a continuous score, coded using 0,1, and 2 for normal, atypical, and not detected RSNs, respectively. The ordinal scores of 0,1,2, and 3 representing normal, mildly abnormal, moderately abnormal, and severely abnormal, respectively, were used for EEG, a-MRI, MRS, and task-fMRI tests. These numeric scores were also used to assess pairwise agreement between modalities using the Spearman rank correlation coefficient. All statistical tests were 2-sided, with statistical significance and CI adjusted using a Bonferroni correction to account for multiple test comparisons for each modality (adjusted threshold of *P* < .006 for 8 or 9 comparisons per modality and adjusted threshold of *P* < .002 for 22 pairwise comparisons among modalities).

## 3 Results

Subjects (N = 40) had a mean (standard deviation) gestational age of 37.8 (2.6) weeks, and included 25 (63%) males, 27 (68%) with HIE, and no RS-related safety events. Three (8%) subjects died prior to discharge and one subject was lost to follow-up. Among the 36 (90%) subjects followed, the median age (interquartile range) at outpatient follow-up visit was 31.6 (13.9, 37.6) weeks. Findings of presentation, neurological exam, consciousness, RS, a-MRI, task-fMRI, MRS, EEG, and outcomes during the acute period are shown in Table 1 and an example of normal versus atypical RSNs is shown in Fig. 1.

All association analyses between tests and clinical factors/outcomes are presented in the supplementary material (eTables 1–8), whilst Tables 2 and Table 3 present those associations showing significance after multiple testing correction.

**Table 2:** Associations of Resting-State Networks (RSN) with Acute Factors and Outcomes in Neonates (N = 40).

	**Factor**	**Category**	**RS Categorizations, n (%)**			***P* value ^a^**	**Ordinal/Multinomial Logistic Regression**	
			**0: Normal**	**1: Atypical**	**2: Not Detected**		**Odds Ratio (99.4% CI)**	***P* value ^b^**
**BASAL GANGLIA**	**Outpatient Developmental Delay**^c^	0: Normal 1: Mild delay 2: Moderate delay or focal findings 3: Severe findings 4: Deceased	9 (90) 0 (0) 1 (10) 0 (0) 0 (0)	9 (39) 2 (9) 9 (39) 2 (9) 1 (4)	1 (17) 0 (0) 0 (0) 3 (50) 2 (33)	0.002**	14.5 (2.00, 105)	0.0002**
	**Outpatient Motor-Tone**^c^	0: Normal 1: Mildly increased tone or weakness 2: Moderately increased tone or weakness 3: Severely increased tone or weakness 4: Deceased	8 (80) 1 (10) 1 (10) 0 (0) 0 (0)	9 (39) 2 (9) 9 (39) 2 (9) 1 (4)	1 (17) 0 (0) 0 (0) 3 (50) 2 (33)	0.007*	9.98 (1.72, 57.9)	0.0003**
**FRONTO-PARIETAL**	**Discharge Condition**	0: Normal 1: Mild support 2: Moderate deficits 3: Deceased	14 (61) 8 (35) 1 (4) 0 (0)	5 (38) 7 (54) 0 (0) 1 (8)	0 (0) 1 (25) 1 (25) 2 (50)	0.006**	5.13 (1.22, 21.5)	0.002**
	**Outpatient Developmental Delay**^c^	0: Normal 1: Mild delay 2: Moderate delay or focal findings 3: Severe findings 4: Deceased	14 (64) 1 (5) 5 (23) 2 (9) 0 (0)	5 (38) 1 (8) 4 (31) 2 (15) 1 (8)	0 (0) 0 (0) 1 (25) 1 (25) 2 (50)	0.05*	4.77 (1.21, 18.7)	0.002**
**DEFAULT MODE**	**Neuro Exam**	0: Normal 1: Mildly abnormal 2: Moderately abnormal 3: Severely abnormal	8 (30) 10 (37) 8 (30) 1 (4)	1 (13) 2 (25) 5 (63) 0 (0)	1 (20) 0 (0) 0 (0) 4 (80)	0.002**	1.00 0.57 (0.05, 6.76) 1.28 (0.17, 9.50) 11.8 (0.73, 191)	0.53 0.73 0.01*
	**Discharge Condition**	0: Normal 1: Mild support 2: Moderate deficits 3: Deceased	16 (59) 10 (37) 1 (4) 0 (0)	2 (25) 4 (50) 1 (13) 1 (13)	1 (20) 2 (40) 0 (0) 2 (40)	0.04*	3.72 (1.01, 13.8)	0.006**
**RS-SOZ**	**Outpatient Motor-Tone**^c^	0: Normal 1: Mildly increased tone or weakness 2: Moderately increased tone or weakness 3: Severely increased tone or weakness 4: Deceased	14 (61) 2 (9) 4 (17) 1 (4) 2 (9)	4 (57) 1 (14) 2 (29) 0 (0) 0 (0)	0 (0) 0 (0) 4 (44) 4 (44) 1 (11)	0.007*	3.31 (1.08, 10.1)	0.003**

* *P* value significant at <0.05; ** *P* value < 0.006 (adjusted using Bonferroni correction). ^a^*P* value from Fisher exact test. ^b^*P* value from ordinal/multinomial logistic regression. ^c^ One patient lost to follow-up. Abbreviations: RS-SOZ, resting-state seizure onset zone or abnormal findings concerning for seizure related pathology; RS, resting state.

**Table 3:** Association of Baseline Factors with Acute Tests on Neonates (N = 40).

	**Factor**	**Category**	**Categorizations, n (%)**				***P* value ^a^**	**Ordinal/Multinomial Logistic Regression**	
			**0: Normal**	**1: Mildly Abnormal**	**2: Moderately Abnormal**	**3: Severely Abnormal**		**Odds Ratio** **(99.4% CI)**	***P* value ^b^**
**Anatomical MRI**	**HIE (N = 27)**	1: Mild 2: Moderate 3: Severe	8 (73) 2 (18) 1 (9)	5 (63) 3 (38) 0 (0)	1 (100) 0 (0) 0 (0)	0 (0) 2 (29) 5 (71)	0.002**	3.54 (1.18, 10.7)	0.002**
	**Neuro Exam**	0: Normal 1: Mildly abnormal 2: Moderately abnormal 3: Severely abnormal	7 (47) 6 (40) 1 (7) 1 (7)	1 (9) 1 (9) 9 (82) 0 (0)	1 (20) 3 (60) 1 (20) 0 (0)	1 (11) 2 (22) 2 (22) 4 (44)	0.0002**	2.14 (1.01, 4.56)	0.006**
	**Outpatient Developmental Delay**^c^	0: Normal 1: Mild delay 2: Moderate delay or focal findings 3: Severe findings 4: Deceased	10 (71) 1 (7) 1 (7) 2 (14) 0 (0)	7 (64) 0 (0) 2 (18) 1 (9) 1 (9)	0 (0) 1 (20) 3 (60) 1 (20) 0 (0)	2 (22) 0 (0) 4 (44) 1 (11) 2 (22)	0.02*	2.15 (0.99, 4.67)	0.007*
	**Seizure**^c,d^	0: No 1: Yes	12 (86) 2 (14)	7 (70) 3 (30)	3 (60) 2 (40)	1 (14) 6 (86)	0.02*	1.0 2.94 (1.02, 8.43)	0.005**
**EEG**			**0: Normal**	**1: Mild Background**	**2: Seizure**	**3: Flat**			
	**Seizure ^c^**^,d^	0: No 1: Yes	2 (100) 0 (0)	18 (86) 3 (14)	2 (17) 10 (83)	0 (0) 0 (0)	0.0001**	1.00 30.4 (2.02, 458)	0.0005**

* *P* value < 0.05; ** *P* value < 0.006 (adjusted using Bonferroni correction); ^a^*P* value from Fisher exact test; ^b^*P* value from ordinal/multinomial logistic regression. ^c^ One patient lost to follow-up. ^d^ Three patients died before discharge. Abbreviations: HIE, hypoxic ischemic encephalopathy; EEG, electroencephalogram; MRI, magnetic resonance imaging.

After multiple testing correction, associations remaining significant for RSNs were: BG with outpatient developmental delay (OR, 14.5; 99.4% CI, 2.00–105; *P* < .001) and motor weakness/tone (OR, 9.98; 99.4% CI, 1.72–57.9; *P* < .001); Lang/FP with discharge condition (OR, 5.13; 99.4% CI, 1.22–21.5; *P* = .002) and outpatient developmental delay (OR, 4.77; 99.4% CI, 1.21–18.7; *P=*.002); DMN with discharge condition (OR, 3.72; 99.4% CI, 1.01–13.8; *P=*.006) and neurological exam (*P = .*002 (FE); OR, 11.8; 99.4% CI, 0.73–191; *P = .*01(OLR)); and RS-SOZ with motor weakness/tone at follow-up (OR, 3.31; 99.4% CI, 1.08–10.1; *P=*.003) (Table 2).

Among the non-RS diagnostics (Table 3), only a-MRI was associated with neurological exam (OR, 2.14; 99.4% CI, 1.01–4.56; *P* < .001) after multiple testing correction. Outpatient seizure concern was associated with both a-MRI (OR, 2.94; 99.4% CI, 1.02–8.43; *P* = .005) and more strongly with EEG (OR, 30.4; 99.4% CI, 2.02–458; *P* < .001) (Table 3).

Cross-modality correlations significant after multiple testing were detected for a-MRI with EEG (CC, 0.52; 99.8% CI, 0.07–0.80; *P* < .001) and MRS (CC, 0.74; 99.8% CI, 0.22–0.93; *P* < .001) (Table 4). While not meeting multiple testing correction criteria, RSN correlation with other diagnostic modalities were suggested only between Lang/FP and task-fMRI, and RS-SOZ with a-MRI (Table 4).

**Table 4:** Spearman Correlation Coefficient and 99.8% Confidence Intervals (CI) between Resting-State Networks (RSN) and Other Test Modalities.

	Spearman Rank Correlation Coefficient (99.8% CI) *P value*						
Test Modalities	RSN				Acute Test Modalities		
	BG	Lang/FP	DMN	RS-SOZ	EEG	Anatomical MRI	Task-fMRI
EEG	0.22 (−0.28, 0.63) *P = 0.17*	0.20 (−0.29, 0.62) *P = 0.21*	0.21 (−0.29, 0.62) *P = 0.19*	0.20 (−0.30, 0.62) *P = 0.21*	–		–
Anatomical MRI	0.18 (−0.32, 0.60) *P = 0.27*	0.27 (−0.34, 0.58) *P = 0.08*	0.15 (−0.34, 0.58) *P = 0.34*	0.44 (−0.04, 0.75) *P = 0.004*	0.52 (0.07, 0.80) P = 0.0004*	–	–
Task-fMRI	0.31 (−0.22, 0.69) *P = 0.07*	0.46 (−0.32, 0.63) *P = 0.004*	0.20 (−0.32, 0.63) *P = 0.23*	−0.12 (−0.58, 0.39) *P = 0.47*	0.25 (−0.28, 0.67) *P = 0.14*	0.38 (−0.14, 0.73) *P = 0.02*	*–*
MRS	0.02 (−0.61, 0.64) *P = 0.93*	0.33 (−*0.49, 0.72)* *P = 0.14*	0.19 (−0.49, 0.72) *P = 0.41*	0.18 (−0.50, 0.72) *P = 0.43*	0.37 (−0.32, 0.81) *P = 0.09*	0.74 (0.22, 0.93) P= <0.0001*	0.57 (−0.12, 0.89) *P = 0.008*

* *P* at < 0.002 meeting multiple testing criteria. Abbreviations: EEG, electroencephalogram; MRI, magnetic resonance imaging; fMRI, functional MRI; MRS, magnetic resonance spectroscopy; BG, basal ganglia network; Lang/FP, language and/or frontoparietal network; DMN, default mode network; RS-SOZ, resting-state seizure onset zone or abnormal findings concerning for seizure related pathology; RSN, resting-state network.

Among the three patients who died, BG, Lang/FP, and DMN were atypical in one and not detected in two patients (Fig. 1). The three that died while in the hospital had insults due to traumatic brain injury (TBI), severe HIE, and placental abruption. The first two neonates (TBI and severe HIE) had no detectable RSNs. The first’s exam progressed to findings consistent with brain death, (Fig. 1) although apnea and cold caloric tests were not performed. In all three, the medical condition was determined to be terminal or futile after RS, and families elected withdrawal of life sustaining therapy. Associations with mortality were stronger for RS (though not significant after correction for multiple testing) than for a-MRI, task-fMRI, MRS, and EEG (eTables 1–8). Associations with consciousness were suggested and comparable for Lang/FP, MRS, DMN, and a-MRI (eTables 1–8).

## 4 Discussion

RSN detection indicated that good signal quality was obtained safely in this neonatal intensive care unit ABI population, thus, worthy of consideration for future patient care. In this study, RS was consistent with prior findings of networks emerging before birth (Doria et al., 2010) and increasing rapidly during this early developmental period (Gao et al., 2015, Gao et al., 2011).

In this cohort of neonates with common ABI etiologies, integrated brain network function via RS was associated with neurological exam and outcomes, suggesting the connectivity degradation was physiological and related to the injury, rather than causes such as the theorized early developmental RSN poor signal, ICU-related therapies, or artifact. These RS findings, pointing to ABI impacting the neural network function and plasticity, are consistent with results from other modalities evaluating adults and children with ABI (Smyser and Neil, 2015).

### 4.1 Incremental outcomes

The *incremental* degradation of RSN connectivity was associated with worsened *outcomes, and findings specific to different networks.* The most severe RSN finding, lack of either the Lang/FP or DMN, made it more likely that upon discharge, there would be severely abnormal exam findings or death. Similarly, worse BG and RS-SOZ were related to marked outpatient developmental delay or motor delay, respectively, or death. Incrementally worse a-MRI was associated with *worsening* acute factors, and *overall* outpatient development at follow-up. This difference may be due to the lack of blinding with cross-informed sub-specialist interpretations in the acute period being greater with more substantiated tests. However, the association with outcomes from these prospectively documented clinical notes may be informative, as suggested by Linke et al. (2018).

### 4.2 Developmental outcomes

The BG and RS-SOZ were associated with motor outcome, suggesting specific insight into early motor skills and correspondence to subcortical-dominated HIE neonatal pathology (Douglas-Escobar and Weiss, 2015). Correspondingly, the spatially broader and cortical RSNs, Lang/FP and DMN, had association with more generalized developmental outcomes. Supportively, FP is one of the early networks detected, as Doria et al. found FP present by term (Doria et al., 2010), and may be predictive of individually unique cognitive capacities (Wang et al., 2021). This may help explain the cognitive and other cortically localized outcomes known to occur after HIE, though longer follow up is indicated (Douglas-Escobar and Weiss, 2015, Pouppirt et al., 2021, van Kooij et al., 2010).

### 4.3 Acute EEG and seizure outcomes

Only the EEG and a-MRI were specifically related to seizure outcomes, whereas the RS-SOZ and seizure outcome association was weaker. Further, the RS-SOZ was *not* associated with acute EEG. The reasons for this could be multifactorial. (1) The study design in determination of seizure outcome was weak, as it was based largely on parent description of repeat events which can be subtle and difficult to discern in neonates, or practitioners’ style of continued anti-seizure management at the time of follow up visit. (2) The seizure, captured on EEG and often clinically resolved by the time of RS, may reflect a true phenomenon of disparately timed tests, capturing the actual evolution of the brain condition in ABI. Supportively, most ABI seizures occur 0–72 h after insult (Lynch et al., 2015), whereas RS capture was *after* 72 h. (3) Conversely, negative EEG could possibly still occur despite deep-source seizures, whereas RS-SOZ has no such spatial limitation. This may explain why the RS-SOZ unadjusted association was highest with a-MRI and subsequent developmental delay and motor assessment (supplementary material (eTables 4) (Lopes et al., 2012, Wurina et al., 2012).

### 4.4 Acute exam, consciousness, and mortality

We demonstrate consistency across exams and tests, which may affect withdrawal of life sustaining therapy and suggests potential for impact on acute-period care. As such, the minimal integrated brain network function for survival and meaningful brain recovery is a key factor. Unadjusted associations suggest that the minimum integrated brain network function by RS for *survival* may be above complete lack of RSN detection, or lack of Lang/FP detection, and subtler findings may change due to neuroplasticity (Boerwinkle et al., 2018a, Boerwinkle et al., 2018b, Boerwinkle et al., 2019a). Also, re-emergence of RSNs is unknown in ABI, though occurs in the anesthetized. This is unknown in neonates, and therefore, is a limitation of care with ethical implications. The Lang/FP connection to mortality could be reflective of this network combination covering a very broad spatial territory of parenchyma. This may mean a large portion of the brain is in the irreversible cell-death cascade, which can trigger a more systemic destabilization, and death. Otherwise, RSN and a-MRI associated outcomes included death, suggesting the linkage to systemic health and death. Thus, serial longitudinal RS after ABI is indicated for evaluation of re-emergence and increased minimal integrated brain network function definition.

The DMN was the only network with statistically significant *overall* association with the acute neurologic exam, which included evaluation of consciousness, and was defined by responsiveness and eye opening to environmental stimuli. This suggests that markers of consciousness by this definition in neonates with ABI across the full severity range may be related to ongoing supratentorial DMN activity, rather than a mere brainstem-mediated phenomenon. This network outcome association is supported by prior work: Lang/FP (Boly et al., 2009, Guo et al., 2016) and DMN (Kondziella et al., 2017, Threlkeld et al., 2018) in other ABI populations, which were associated with acute neurological findings. Comparatively, among the non-RSN tests, a-MRI was also associated with the neurological exam.

*HIE severity* was associated with a-MRI, but not RSNs. This could be due to the study design in which the HIE severity was ultimately determined at discharge after all diagnostics and acute exams. Thus, HIE severity was not blinded, and may have been largely based on the a-MRI findings. A blinded prospective study would shed light on the question.

### 4.5 Cross-modality observations

The cross-modality severity consistency has supportive and unique results in this real-world all-ABI-comer neonatal population. Namely, the BG, a-MRI, and MRS rates of being atypical were commensurate. Contrastingly, the cortical RSNs had similar but lower atypical rates. Thus, subcortical, more so than cortical, network abnormality is as expected in an HIE-rich cohort (de Vries and Groenendaal, 2010). Notably, non-RS tests had higher abnormality rates, yet the RSNs had higher frequency of association with outcomes, suggesting unique developmental information from RSNs.

Inter-modality disparities are expected and reflect different physiological signals. Thus, the higher rate of cortical RSN normality is in-line with task-fMRI, measuring cortical motor function. Similarly, MRS, measuring deep-brain location metabolism, was more often abnormal in this HIE-rich population. Hence, the disparities are consistent and triangulate on different and localizing aspects of the ABI etiology.

### 4.6 Limitations and further study

A prospective approach with standardized exams and outcome measures is needed. Similarly, validated neonatal consciousness and other acute factor and outcome measures are necessary for mixed pathology/full-range severity populations. It is of great interest to determine if increasing burden of key atypical findings is related to outcome. Our preliminary data suggests that the burden of common multiple atypical findings such as lack of recognizable detection of key modulating and cognition networks in individual neonates may cluster in those with the worst outcomes and is of great interest for future study. RS requires interpretation —prompting the blinded study design. Further means of minimizing bias by developing automated RSN categorization may help (Chakraborty et al., 2020, Griffanti et al., 2017, Griffanti et al., 2014).

In neonates with ABI, network pathology localization is not well-characterized and data-driven approaches, such as ICA may best be suited for localization of network pathology. ICA has level 1 evidence for diagnostic testing (OCEBM Levels of Evidence Working Group) in children and adults for characterizing normal and pathological networks in DRE and improving outcomes (Chakraborty et al., 2020), however, method comparison may be of value.

## 5 Conclusions

This study provides level 3 evidence (OCEBM Levels of Evidence Working Group) that RSNs are associated with outcomes in neonates with ABI. Specific networks’ degradation was associated with higher severity outcomes. The basal ganglia and seizure onset zone networks were associated with motor outcomes. The broad language/cognitive region networks were associated with developmental delay. Discharge condition, which includes mortality, was associated with default mode and language/cognitive networks. Lack of all studied networks only occurred in those who did not survive.

### CRediT authorship contribution statement

**Varina L. Boerwinkle:** Conceptualization, Methodology, Software, Validation, Formal analysis, Investigation, Resources, Data curation, Writing – original draft, Writing – review & editing, Visualization, Supervision, Project administration, Funding acquisition. **Bethany Sussman:** Software, Validation, Formal analysis, Investigation, Data curation, Writing – original draft, Writing – review & editing, Visualization. **Iliana Manjón:** Validation, Formal analysis, Investigation, Data curation, Writing – original draft, Writing – review & editing, Visualization. **Lucia Mirea:** Validation, Formal analysis, Investigation, Resources, Writing – original draft, Writing – review & editing, Visualization. **Saher Suleman:** Investigation, Data curation, Writing – original draft, Writing – review & editing. **Sarah N. Wyckoff:** Software, Validation, Investigation, Formal analysis, Data curation, Writing – original draft, Writing – review & editing, Visualization. **Alexandra Bonnell:** Investigation, Data curation, Writing – original draft, Writing – review & editing. **Andrew Orgill:** Validation, Formal analysis, Writing – original draft, Writing – review & editing. **Deborah Tom:** Validation, Resources, Writing – original draft, Writing – review & editing.

## Declaration of Competing Interest

The authors declare that they have no known competing financial interests or personal relationships that could have appeared to influence the work reported in this paper.
