# Supplementary material for: Association of network connectivity via resting state functional MRI with consciousness, mortality, and outcomes in neonatal acute brain injury
Source: Neuroimage Clin. 2022 Feb 9;34:102962. doi: 10.1016/j.nicl.2022.102962 (PMC8851268; doi:10.1016/j.nicl.2022.102962)
Supplement: Supplementary Data 1 [file mmc1.docx]

**eFigure 1. Algorithm for independent component classification. Figure updated and modified from Boerwinkle et al., 2017 (**Copyright © Varina L. Boerwinkle et al. 2017; Published by Mary Ann Liebert, Inc)**.** Abbreviations: CSF, cerebral spinal fluid; EPI, resting-state MRI sequence; SOZ, seizure onset zone; GM, grey matter; IC, independent component; RSN, resting-state network; WM, white matter.

**eTable 1: Association of Resting State Basal Ganglia (RS-BG) with Outcomes.**

| **Factor** | **RS-Basal Ganglia** | | | **P**  **value ^a^** | **Ordinal/Multinomial**  **Logistic Regression** | |
| --- | --- | --- | --- | --- | --- | --- |
|  | **0: Normal**  **(N=10)** | **1: Atypical**  **(N=24)** | **2: Not Detected**  **(N=6)** |  | **Odds Ratio**  **(99.4% CI)** | **P**  **value ^b^** |
| **HIE**, N (%)  0: No  1: Yes | 4 (40)  6 (60) | 8 (33)  16 (67) | 1 (17)  5 (83) | 0.72 | 1.00  1.66 (0.36, 7.70) | -  0.36 |
| **HIE**, N (%)  1: Mild  2: Moderate  3: Severe | 6 (100)  0 (0)  0 (0) | 6 (38)  6 (38)  4 (25) | 2 (40)  1 (20)  2 (40) | 0.09 | 4.59 (0.68, 31) | 0.03* |
| **Neuro Exam**, N (%)  0: Normal  1: Mildly abnormal  2: Moderately abnormal  3: Severely abnormal | 2 (20)  4 (40)  4 (40)  0 (0) | 7 (29)  8 (33)  8 (33)  1 (4) | 1 (17)  0 (0)  1 (17)  4 (67) | 0.02* | 1.00  0.44 (0.04, 4.30)  0.62 (0.07, 5.77)  32.1 (0.73, >99) | -  0.32  0.56  0.01* |
| **Consciousness (Day 0-5)**, N (%)  0: Normal  1: Irritable but arouses easily  2: Wakes up only to painful stimulation  3: Coma  4: Episodically arousable | 2 (20)  5 (50)  2 (20)  1 (10)  0 (0) | 6 (25)  10 (42)  7 (29)  0 (0)  1 (4) | 1 (17)  1 (17)  2 (33)  2 (33)  0 (0) | 0.32 | 1.67 (0.46, 6.07) | 0.27 |
| **Death**, N (%)  0: No  1: Yes | 10 (100)  0 (0) | 23 (96)  1 (4) | 4 (67)  2 (33) | 0.10 | 1.00  12.62 (0.38, 414) | -  0.05* |
| **Discharge Condition**, N (%)  0: Normal  1: Mild support  2: Moderate deficits  3: Deceased | 5 (50)  5 (50)  0 (0)  0 (0) | 13 (54)  10 (42)  0 (0)  1 (4) | 1 (17)  1 (17)  2 (33)  2 (33) | 0.02* | 1.00  0.87 (0.15, 4.95)  >99 (<0.01, >99)  22.9 (0.47, >99) | -  0.83  0.99  0.03* |
| **Outpatient Development**, N (%)  0: Normal  1: Mild delay  2: Moderate delay or focal finding on exam  3: Severe findings  4: Deceased | 9 (90)  0 (0)  1 (10)  0 (0)  0 (0) | 9 (39)  2 (9)  9 (39)  2 (9)  1 (4) | 1 (17)  0 (0)  0 (0)  3 (50)  2 (33) | 0.002** | 14.5 (2.0, 105) | 0.0002** |
| **Outpatient Motor-Tone**, N (%)  0: Normal  1: Mildly increased tone or weakness  2: Moderately increased tone or weakness  3: Severely increased tone or weakness  4: Deceased | 8 (80)  1 (10)  1 (10)  0 (0)  0 (0) | 9 (39)  2 (9)  9 (39)  2 (9)  1 (4) | 1 (17)  0 (0)  0 (0)  3 (50)  2 (33) | 0.007 | 9.98 (1.72, 57.9) | 0.0003** |

* P value significant at <.05 (not adjusted using Bonferroni correction). ** P value significant or marginally significant at <.006 (adjusted using Bonferroni correction). ^a^ P value from Fisher exact test. ^b^ P value from ordinal/multinomial logistic regression.

**eTable 2: Association of Resting State (RS) Language and/or Frontoparietal (Lang-FP) with Outcomes**

| **Factor** | **RS-Language and/or**  **Frontoparietal** | | | **P value ^a^** | **Ordinal/Multinomial**  **Logistic Regression** | |
| --- | --- | --- | --- | --- | --- | --- |
|  | 0: Normal  (N=23) | 1: Atypical  (N=13) | 2: Not detected  (N=4) |  | **Odds Ratio**  **(99.4% CI)** | **P value ^b^** |
| **HIE**, N (%)  0: No  1: Yes | 7 (30)  16 (70) | 4 (31)  9 (69) | 2 (50)  2 (50) | 0.78 | 1.00  0.75 (0.19, 2.91) | -  0.56 |
| **HIE**, N (%)  1: Mild  2: Moderate  3: Severe | 11 (69)  2 (13)  3 (19) | 3 (33)  4 (44)  2 (22) | 0 (0)  1 (50)  1 (50) | 0.12 | 3.13 (0.58, 17.0) | 0.06 |
| **Neuro Exam**, N (%)  0: Normal  1: Mildly abnormal  2: Moderately abnormal  3: Severely abnormal | 7 (30)  8 (35)  7 (30)  1 (4) | 3 (23)  3 (23)  5 (38)  2 (15) | 0 (0)  1 (25)  1 (25)  2 (50) | 0.35 | 2.72 (0.77, 9.56) | 0.03* |
| **Consciousness (Day 0-5)**, N (%)  0: Normal  1: Irritable but arouses easily  2: Wakes up only to painful stimulation  3: Coma  4: Episodically arousable | 7 (30)  9 (39)  6 (26)  1 (4)  0 (0) | 2 (15)  6 (46)  4 (30)  0 (0)  1 (8) | 0 (0)  1 (25)  1 (25)  2 (50)  0 (0) | 0.19 | 2.67 (0.76, 9.39) | 0.03* |
| **Death**, N (%)  0: No  1: Yes | 23 (100)  0 (0) | 12 (92)  1 (8) | 2 (50)  2 (50) | 0.008 | 1.00  16.4 (0.54, 498) | -  0.02* |
| **Discharge Condition**, N (%)  0: Normal  1: Mild support  2: Moderate deficits  3: Deceased | 14 (61)  8 (35)  1 (4)  0 (0) | 5 (38)  7 (54)  0 (0)  1 (8) | 0 (0)  1 (25)  1 (25)  2 (50) | 0.006** | 5.13 (1.22, 21.51) | 0.002** |
| **Outpatient Development**, N (%)  0: Normal  1: Mild delay  2: Moderate delay or focal finding on exam  3: Severe findings  4: Deceased | 14 (64)  1 (5)  5 (23)  2 (9)  0 (0) | 5 (38)  1 (8)  4 (31)  2 (15)  1 (8) | 0 (0)  0 (0)  1 (25)  1 (25)  2 (50) | 0.05* | 4.77 (1.21, 18.7) | 0.002** |
| **Outpatient Motor-Tone**, N (%)  0: Normal  1: Mildly increased tone or weakness  2: Moderately increased tone or weakness  3: Severely increased tone or weakness  4: Deceased | 12 (55)  2 (9)  6 (27)  2 (9)  0 (0) | 5 (38)  1 (8)  4 (31)  2 (15)  1 (8) | 1 (25)  0 (0)  0 (0)  1 (25)  2 (50) | 0.16 | 3.35 (0.92, 12.1) | 0.01* |

* P value significant at <.05 (not adjusted using Bonferroni correction). ** P value significant or marginally significant at <.006 (adjusted using Bonferroni correction). ^a^ P value from Fisher exact test; ^b^ P value from ordinal/multinomial logistic regression.

**eTable 3: Association of Resting State (RS) Default Mode Network with Outcomes**

| **Factor** | **RS-Default Mode Network** | | | **P value ^a^** | **Ordinal/Multinomial**  **Logistic Regression** | |
| --- | --- | --- | --- | --- | --- | --- |
|  | **0: Normal**  **(N=27)** | **1: Atypical**  **(N=8)** | **2: Not Detected**  **(N=5)** |  | **Odds Ratio**  **(99.4% CI)** | **P value ^b^** |
| **HIE**, N (%)  0: No  1: Yes | 9 (33)  18 (67) | 2 (25)  6 (75) | 2 (40)  3 (60) | 1.00 | 1.00  0.97 (0.26, 3.58) | -  0.94 |
| **HIE**, N (%)  1: Mild  2: Moderate  3: Severe | 12 (67)  3 (17)  3 (17) | 1 (17)  4 (67)  1 (17) | 1 (33)  0 (0)  2 (67) | 0.03* | 3.32 (0.68, 16.3) | 0.04* |
| **Neuro Exam**, N (%)  0: Normal  1: Mildly abnormal  2: Moderately abnormal  3: Severely abnormal | 8 (30)  10 (37)  8 (30)  1 (4) | 1 (13)  2 (25)  5 (63)  0 (0) | 1 (20)  0 (0)  0 (0)  4 (80) | 0.002** | 1.00  0.57 (0.05, 6.76)  1.28 (0.17, 9.50)  11.8 (0.73, 191) | -  0.53  0.73  0.01* |
| **Consciousness (Day 0-5)**, N (%)  0: Normal  1: Irritable but arouses easily  2: Wakes up only to painful stimulation  3: Coma  4: Episodically arousable | 7 (26)  12 (44)  7 (26)  1 (4)  0 (0) | 2 (25)  2 (25)  3 (38)  0 (0)  1 (12) | 0 (0)  2 (40)  1 (20)  2 (40)  0 (0) | 0.16 | 2.29 (0.71, 7.45) | 0.05* |
| **Death**, N (%)  0: No  1: Yes | 27 (100)  0 (0) | 7 (88)  1 (13) | 3 (60)  2 (40) | 0.01 | 1.00  10.1 (0.56, 180) | -  0.03* |
| **Discharge Condition**, N (%)  0: Normal  1: Mild support  2: Moderate deficits  3: Deceased | 16 (59)  10 (37)  1 (4)  0 (0) | 2 (25)  4 (50)  1 (13)  1 (13) | 1 (20)  2 (40)  0 (0)  2 (40) | 0.04* | 3.72 (1.01, 13.8) | 0.006** |
| **Outpatient Development**, N (%)  0: Normal  1: Mild delay  2: Moderate delay or focal finding on exam  3: Severe findings  4: Deceased | 15 (58)  1 (4)  8 (31)  2 (8)  0 (0) | 3 (38)  0 (0)  2 (25)  2 (25)  1 (13) | 1 (20)  1 (20)  0 (0)  1 (20)  2 (40) | 0.02* | 1.00  4.43 (0.28, 70.6)  0.77 (0.08, 7.30)  3.25 (0.42, 24.9)  15.6 (0.61, 396) | -  0.14  0.75  0.11  0.02* |
| **Outpatient Motor-Tone**, N (%)  0: Normal  1: Mildly increased tone or weakness  2: Moderately increased tone or weakness  3: Severely increased tone or weakness  4: Deceased | 15 (58)  1 (4)  8 (31)  2 (8)  0 (0) | 2 (25)  1 (13)  2 (25)  2 (25)  1 (13) | 1 (20)  1 (20)  0 (0)  1 (20)  2 (40) | 0.02* | 1.00  5.38 (0.43, 67.2)  0.90 (0.08, 9.41)  3.92 (0.45, 34.2)  19.4 (0.66, 567) | -  0.07  0.90  0.08  0.02* |

* P value significant at <.05 (not adjusted using Bonferroni correction). ** P value significant or marginally significant at <.006 (adjusted using Bonferroni correction). ^a^ P value from Fisher exact test. ^b^ P value from ordinal/multinomial logistic regression.

**eTable 4: Association of Resting State (RS) Seizure Onset Zone or Abnormal Findings Concerning for Seizure with Outcomes**

| **Factor** | **RS-Seizure Onset Zone or Abnormal Findings Concerning for Seizure** | | | **P value ^a^** | **Ordinal/Multinomial**  **Logistic Regression** | |
| --- | --- | --- | --- | --- | --- | --- |
|  | **0: Normal**  **(N=24)** | **1: Some Concern for Seizure (N=7)** | **2: High Concern for Seizure**  **(N=9)** |  | **Odds Ratio**  **(99.4 CI)** | **P value ^b^** |
| **HIE**, N (%)  0: No  1: Yes | 8 (33)  16 (67) | 2 (29)  5 (71) | 3 (33)  6 (67) | 1.00 | 1.00  1.02 (0.32, 3.22) | -  0.96 |
| **HIE**, N (%)  1: Mild  2: Moderate  3: Severe | 11 (69)  3 (19)  2 (13) | 1 (20)  1 (20)  3 (60) | 2 (33)  3 (50)  1 (17) | 0.09 | 1.92 (0.54, 6.81) | 0.15 |
| **Neuro Exam**, N (%)  0: Normal  1: Mildly abnormal  2: Moderately abnormal  3: Severely abnormal | 7 (30)  9 (38)  6 (25)  2 (8) | 1 (14)  2 (29)  3 (43)  1 (14) | 2 (22)  1 (11)  4 (44)  2 (22) | 0.64 | 1.79 (0.66, 4.90) | 0.10 |
| **Consciousness (Day 0-5)**, N (%)  0: Normal  1: Irritable but arouses easily  2: Wakes up only to painful stimulation  3: Coma  4: Episodically arousable | 5 (21)  12 (50)  5 (21)  2 (8)  0 (0) | 2 (29)  1 (14)  3 (43)  1 (14)  0 (0) | 2 (22)  3 (33)  3 (33)  0 (0)  1 (11) | 0.46 | 1.00  0.69 (0.16, 3.04)  1.23 (0.28, 5.42)  0.55 (0.04, 8.71)  >99 (<0.01, >99) | -  0.48  0.70  0.54  0.99 |
| **Death**, N (%)  0: No  1: Yes | 22 (92)  2 (8) | 7 (100)  0 (0) | 8 (89)  1 (11) | 1.00 | 1.00  1.07 (0.14, 7.90) | -  0.93 |
| **Discharge Condition**, N (%)  0: Normal  1: Mild support  2: Moderate deficits  3: Deceased | 12 (50)  9 (3)  1 (4)  2 (9) | 4 (57)  3 (43)  0 (0)  0 (0) | 3 (33)  4 (44)  1 (11)  1 (11) | 0.95 | 1.32 (0.47, 3.65) | 0.45 |
| **Outpatient Development**, N (%)  0: Normal  1: Mild delay  2: Moderate delay or focal finding on exam  3: Severe findings  4: Deceased | 14 (61)  1 (4)  5 (22)  1 (4)  2 (9) | 5 (71)  0 (0)  2 (29)  0 (0)  0 (0) | 0 (0)  1 (11)  3 (33)  4 (44)  1 (11) | 0.007* | 2.74 (0.93, 8.06) | 0.009* |
| **Outpatient Motor-Tone**, N (%)  0: Normal  1: Mildly increased tone or weakness  2: Moderately increased tone or weakness  3: Severely increased tone or weakness  4: Deceased | 14 (61)  2 (9)  4 (17)  1 (4)  2 (9) | 4 (57)  1 (14)  2 (29)  0 (0)  0 (0) | 0 (0)  0 (0)  4 (44)  4 (44)  1 (11) | 0.007* | 3.31 (1.08, 10.1) | 0.003** |
| **Concern for Seizure**, N (%)  0: No  1: Yes  (Deceased/lost to follow-up, N=4) | 17 (81)  4 (19) | 3 (43)  4 (57) | 3 (38)  5 (63) | 0.04* | 2.83 (0.78, 10.22) | 0.02* |

* P value significant at <.05 (not adjusted using Bonferroni correction). ** P value significant or marginally significant at <.006 (adjusted using Bonferroni correction). ^a^ P value from Fisher exact test; ^b^ P value from ordinal/multinomial logistic regression.

**eTable 5: Association of Baseline Factors with Anatomical MRI.**

| **Factor** | **Anatomical MRI** | | | | **P value ^a^** | **Ordinal/Multinomial**  **Logistic Regression** | |
| --- | --- | --- | --- | --- | --- | --- | --- |
|  | **0: Normal (N=15)** | **1: Mildly Abnormal (N=11)** | **2: Moderately Abnormal (N=5)** | **3: Severely Abnormal (N=9)** |  | **Odds Ratio**  **(99.4% CI)** | **P**  **value ^b^** |
| **HIE**, N (%)  0: No  1: Yes | 4 (27)  11 (73) | 3 (27)  8 (73) | 4 (80)  1 (20) | 2 (22)  7 (78) | 0.14 | 1.00  0.89 (0.40, 1.96) | -  0.69 |
| **HIE**, N (%)  1: Mild  2: Moderate  3: Severe | 8 (73)  2 (18)  1 (9) | 5 (63)  3 (38)  0 (0) | 1 (100)  0 (0)  0 (0) | 0 (0)  2 (29)  5 (71) | 0.002** | 3.54 (1.18, 10.68) | 0.002** |
| **Neuro Exam**, N (%)  0: Normal  1: Mildly abnormal  2: Moderately abnormal  3: Severely abnormal | 7 (47)  6 (40)  1 (7)  1 (7) | 1 (9)  1 (9)  9 (82)  0 (0) | 1 (20)  3 (60)  1 (20)  0 (0) | 1 (11)  2 (22)  2 (22)  4 (44) | 0.0002** | 2.14 (1.01, 4.56) | 0.006** |
| **Consciousness (Day 0-5)**, N (%)  0: Normal  1: Irritable but arouses easily  2: Wakes up only to painful stimulation  3: Coma  4: Episodically arousable | 5 (33)  8 (53)  2 (13)  0 (0)  0 (0) | 1 (9)  4 (36)  4 (36)  1 (9)  1 (9) | 2 (40)  2 (40)  1 (20)  0 (0)  0 (0) | 1 (11)  2 (22)  4 (44)  2 (22)  0 (0) | 0.30 | 1.70 (0.83, 3.48) | 0.04* |
| **Death**, N (%)  0: No  1: Yes | 15 (100)  0 (0) | 10 (91)  1 (9) | 5 (100)  0 (0) | 7 (78)  2 (22) | 0.14 | 1.00  2.57 (0.47, 13.9) | -  0.12 |
| **Discharge Condition**, N (%)  0: Normal  1: Mild support  2: Moderate deficits  3: Deceased | 8 (53)  6 (40)  1 (7)  0 (0) | 6 (55)  4 (36)  0 (0)  1 (9) | 2 (40)  3 (60)  0 (0)  0 (0) | 3 (3)  3 (3)  1 (11)  2 (22) | 0.72 | 1.48 (0.72, 3.06) | 0.14 |
| **Outpatient Development**, N (%)  0: Normal  1: Mild delay  2: Moderate delay or focal finding on exam  3: Severe findings  4: Deceased | 10 (71)  1 (7)  1 (7)  2 (14)  0 (0) | 7 (64)  0 (0)  2 (18)  1 (9)  1 (9) | 0 (0)  1 (20)  3 (60)  1 (20)  0 (0) | 2 (22)  0 (0)  4 (44)  1 (11)  2 (22) | 0.02* | 2.15 (0.99, 4.67) | 0.007** |
| **Outpatient Motor-Tone**, N (%)  0: Normal  1: Mildly increased tone or weakness  2: Moderately increased tone or weakness  3: Severely increased tone or weakness  4: Deceased | 8 (57)  3 (21)  1 (7)  2 (14)  0 (0) | 7 (64)  0 (0)  2 (18)  1 (9)  1 (9) | 1 (20)  0 (0)  3 (60)  1 (20)  0 (0) | 2 (22)  0 (0)  4 (44)  1 (11)  2 (22) | 0.09 | 1.00  <0.01 (<0.01, >99)  2.73 (0.88, 8.55)  1.42 (0.37, 5.44)  3.80 (0.57, 25.5) | -  0.99  0.02*  0.47  0.05* |
| **Concern for Seizure**, N (%)  0: No  1: Yes  (Deceased/lost to follow-up, N=4) | 12 (86)  2 (14) | 7 (70)  3 (30) | 3 (60)  2 (40) | 1 (14)  6 (86) | 0.02* | 2.94 (1.02, 8.43) | 0.005** |

* P value significant at <.05 (not adjusted using Bonferroni correction). ** P value significant or marginally significant at <.006 (adjusted using Bonferroni correction). ^a^ P value from Fisher exact test; ^b^ P value from ordinal/multinomial logistic regression.

**eTable 6: Association of Baseline Factors with Task-fMRI.**

| **Factor** | **Task-fMRI** | | | **P value ^a^** | **Ordinal/Multinomial**  **Logistic Regression** | |
| --- | --- | --- | --- | --- | --- | --- |
|  | **0: Normal**  **(N=30)** | **2: Moderately Abnormal (N=3)** | **3: Severely Abnormal (N=3)** |  | **Odds Ratio**  **(99.4% CI)** | **P value ^b^** |
| **HIE**, N (%)  0: No  1: Yes | 9 (30)  21 (70) | 1 (33)  2 (77) | 1 (33)  2 (67) | 0.99 | 0.94 (0.34, 2.62) | 0.87 |
| **HIE**, N (%)  1: Mild  2: Moderate  3: Severe | 12 (57)  6 (29)  3 (14) | 0 (0)  0 (0)  2 (100) | 0 (0)  1 (50)  1 (50) | 0.02* | 2.87 (0.72, 11.4) | 0.04* |
| **Neuro Exam**, N (%)  0: Normal  1: Mildly abnormal  2: Moderately abnormal  3: Severely abnormal | 9 (30)  7 (23)  11 (37)  3 (10) | 0 (0)  1 (33)  1 (33)  1 (33) | 0 (0)  1 (33)  1 (33)  1 (33) | 0.57 | 1.64 (0.66, 4.07) | 0.14 |
| **Consciousness (Day 0-5)**, N (%)  0: Normal  1: Irritable but arouses easily  2: Wakes up only to painful stimulation  3: Coma  4: Episodically arousable | 8 (27)  12 (40)  7 (23)  2 (7)  1 (3) | 0 (0)  1 (33)  2 (67)  0 (0)  0 (0) | 0 (0)  1 (33)  1 (33)  1 (33)  0 (0) | 0.55 | 1.67 (0.68, 4.12) | 0.12 |
| **Death**, N (%)  0: No  1: Yes | 28 (93)  2 (7) | 3 (100)  0 (0) | 2 (67)  1 (33) | 0.43 | 1.00  1.66 (0.44, 6.32) | -  0.30 |
| **Discharge Condition**, N (%)  0: Normal  1: Mild support  2: Moderate deficits  3: Deceased | 15 (50)  12 (40)  1 (3)  2 (7) | 1 (33)  2 (67)  0 (0)  0 (0) | 0 (0)  1 (33)  1 (33)  1 (33) | 0.15 | 2.01 (0.77, 5.25) | 0.05* |
| **Outpatient Development**, N (%)  0: Normal  1: Mild delay  2: Moderate delay or focal finding on exam  3: Severe findings  4: Deceased | 16 (55)  1 (3)  6 (21)  4 (14)  2 (7) | 1 (33)  0 (0)  2 (67)  0 (0)  0 (0) | 0 (0)  0 (0)  1 (33)  1 (33)  1 (33) | 0.24 | 1.78 (0.72, 4.38) | 0.08 |
| **Outpatient Motor-Tone**, N (%)  0: Normal  1: Mildly increased tone or weakness  2: Moderately increased tone or weakness  3: Severely increased tone or weakness  4: Deceased | 14 (48)  3 (10)  6 (21)  4 (14)  2 (7) | 2 (67)  0 (0)  1 (33)  0 (0)  0 (0) | 0 (0)  0 (0)  1 (33)  1 (33)  1 (33) | 0.45 | 1.55 (0.64, 3.73) | 0.17 |

* P value significant at <.05 (not adjusted using Bonferroni correction). ** P value significant or marginally significant at <.006 (adjusted using Bonferroni correction). ^a^ P value from Fisher exact test; ^b^ P value from ordinal/multinomial logistic regression.

**eTable 7: Association of Baseline Factors with Magnetic Resonance Spectroscopy (MRS).**

| **Factor** | **MRS** | | | | **P value ^a^** | **Ordinal/Multinomial**  **Logistic Regression** | |
| --- | --- | --- | --- | --- | --- | --- | --- |
|  | **0: Normal (N=15)** | **1: Mildly Abnormal (N=11)** | **2: Moderately Abnormal (N=5)** | **3: Severely Abnormal (N=9)** |  | **Odds Ratio**  **(99.4% CI)** | **P value ^b^** |
| **HIE**, N (%)  0: No  1: Yes | 1 (8)  11 (92) | 0 (0)  5 (100) | 0 (0)  1 (100) | 0 (0)  3 (100) | 1.0 | **-** | **-** |
| **HIE**, N (%)  1: Mild  2: Moderate  3: Severe | 7 (64)  3 (27)  1 (9) | 4 (80)  0 (0)  1 (20) | 0 (0)  1 (100)  0 (0) | 0 (0)  0 (0)  3 (100) | 0.02* | 3.28 (0.82, 13.1) | 0.02* |
| **Neuro Exam**, N (%)  0: Normal  1: Mildly abnormal  2: Moderately abnormal  3: Severely abnormal | 4 (33)  3 (25)  4 (33)  1 (8) | 0 (0)  1 (20)  4 (80)  0 (0) | 0 (0)  1 (100)  0 (0)  0 (0) | 0 (0)  1 (33)  0 (0)  2 (67) | 0.10 | 2.21 (0.70, 6.99) | 0.06 |
| **Consciousness (Day 0-5)**, N (%)  0: Normal  1: Irritable but arouses easily  2: Wakes up only to painful stimulation  3: Coma  4: Episodically arousable | 4 (33)  6 (50)  2 (17)  0 (0)  0 (0) | 0 (0)  1 (20)  3 (60)  1 (20)  0 (0) | 0 (0)  1 (100)  0 (0)  0 (0)  0 (0) | 0 (0)  1 (33)  1 (33)  1 (33)  0 (0) | 0.20 | 2.45 (0.75, 8.02) | 0.04* |
| **Death**, N (%)  0: No  1: Yes | 12 (100)  0 (0) | 5 (100)  0 (0) | 1 (100)  0 (0) | 2 (67)  1 (33) | 0.19 | - | - |
| **Discharge Condition**, N (%)  0: Normal  1: Mild support  2: Moderate deficits  3: Deceased | 7 (58)  5 (42)  0 (0)  0 (0) | 3 (60)  2 (40)  0 (0)  0 (0) | 1 (100)  0 (0)  0 (0)  0 (0) | 1 (33)  1 (33)  0 (0)  1 (33) | 0.52 | 1.47 (0.48, 4.54) | 0.34 |
| **Outpatient Development**, N (%)  0: Normal  1: Mild delay  2: Moderate delay or focal finding on exam  3: Severe findings  4: Deceased | 8 (73)  0 (0)  1 (9)  2 (18)  0 (0) | 3 (60)  0 (0)  1 (20)  1 (20)  0 (0) | 1 (100)  0 (0)  0 (0)  0 (0)  0 (0) | 1 (33)  0 (00)  1 (33)  0 (0)  1 (33) | 0.56 | 1.00  -  1.84 (0.38, 9.05)  0.63 (0.05, 8.52)  >99 (<0.01, >99) | -  -  0.29  0.62  1.00 |
| **Outpatient Motor-Tone**, N (%)  0: Normal  1: Mildly increased tone or weakness  2: Moderately increased tone or weakness  3: Severely increased tone or weakness  4: Deceased | 7 (64)  1 (9)  1 (9)  2 (18)  0 (0) | 3 (60)  0 (0)  1 (20)  1 (20)  0 (0) | 1 (100)  0 (0)  0 (0)  0 (0)  0 (0) | 1 (33)  0 (0)  1 (33)  0 (0)  1 (33) | 0.76 | 1.00  <0.01 (<0.01, >99)  1.76 (0.35, 8.74)  0.58 (0.04, 8.29)  >99 (<0.01, >99) | -  1.0  0.33  0.57  1.00 |

* P value significant at <.05 (not adjusted using Bonferroni correction). ** P value significant or marginally significant at <.006 (adjusted using Bonferroni correction). ^a^ P value from Fisher exact test; ^b^ P value from ordinal/multinomial logistic regression.

**eTable 8: Association of Baseline Factors with electroencephalogram (EEG).**

| **Factor** | **EEG** | | | | **P value ^a^** | **Ordinal/Multinomial**  **Logistic Regression** | |
| --- | --- | --- | --- | --- | --- | --- | --- |
|  | **0: Normal**  **(N=3)** | **1: Mild Background Abnormality Only**  **(N=22)** | **2: Seizure**  **(N=13)** | **3: Flat**  **(N=1)** |  | **Odds Ratio**  **(99.4% CI)** | **P value ^b^** |
| **HIE**, N (%)  0: No  1: Yes | 0 (0)  3 (100) | 7 (32)  15 (68) | 4 (31)  9 (69) | 1 (100)  0 (0) | 0.39 | 0.51 (0.11, 2.36) | 0.22 |
| **HIE**, N (%)  1: Mild  2: Moderate  3: Severe | 3 (100)  0 (0)  0 (0) | 8 (53)  5 (33)  2 (13) | 3 (33)  2 (22)  4 (44) | 0 (0)  0 (0)  0 (0) | 0.29 | 4.56 (0.66, 31.32 | 0.03* |
| **Neuro Exam**, N (%)  0: Normal  1: Mildly abnormal  2: Moderately abnormal  3: Severely abnormal | 2 (67)  0 (0)  1 (33)  0 (0) | 6 (27)  7 (32)  7 (32)  2 (9) | 2 (15)  4 (31)  5 (39)  2 (15) | 0 (0)  0 (0)  0 (0)  1 (100) | 0.55 | 2.75 (0.73, 10.3) | 0.04* |
| **Consciousness (Day 0-5)**, N (%)  0: Normal  1: Irritable but arouses easily  2: Wakes up only to painful stimulation  3: Coma  4: Episodically arousable | 2 (67)  0 (0)  1 (33)  0 (0)  0 (0) | 5 (23)  9 (41)  6 (27)  1 (5)  1 (5) | 2 (15)  6 (46)  4 (31)  1 (8)  0 (0) | 0 (0)  0 (0)  0 (0)  1 (100)  0 (0) | 0.43 | 2.13 (0.59, 7.69) | 0.11 |
| **Death**, N (%)  0: No  1: Yes | 3 (100)  0 (0) | 21 (95)  1 (5) | 12 (92)  1 (8) | 0 (0)  1 (100) | 0.14 | 1.00  7.03 (0.31, 161) | -  0.09 |
| **Discharge Condition**, N (%)  0: Normal  1: Mild support  2: Moderate deficits  3: Deceased | 1 (33)  2 (67)  0 (0)  0 (0) | 12 (55)  8 (36)  1 (5)  1 (5) | 6 (46)  5 (38)  1 (8)  1 (8) | 0 (0)  0 (0)  0 (0)  1 (100) | 0.52 | 1.66 (0.44, 6.23) | 0.29 |
| **Outpatient Development**, N (%)  0: Normal  1: Mild delay  2: Moderate delay or focal finding on exam  3: Severe findings ^d^  4: Deceased | 1 (50)  0 (0)  0 (0)  1 (50)  0 (0) | 14 (64)  1 (5)  4 (18)  2 (9)  1 (5) | 3 (23)  1 (8)  6 (46)  2 (15)  1 (8) | 0 (0)  0 (0)  0 (0)  0 (0)  1 (100) | 0.09 | 3.46 (0.79, 15.2) | 0.02* |
| **Outpatient Motor-Tone**, N (%)  0: Normal  1: Mildly increased tone or weakness  2: Moderately increased tone or weakness  3: Severely increased tone or weakness  4: Deceased | 1 (50)  0 (0)  0 (0)  1 (50)  0 (0) | 14 (64)  2 (9)  3 (14)  2 (9)  1 (5) | 3 (23)  0 (0)  7 (54)  2 (15)  1 (8) | 0 (0)  0 (0)  0 (0)  0 (0)  0 (0) | 0.02* | 1.00  0.65 (0.01, 38.8)  7.06 (0.75, 66.5)  1.39 (0.10, 20.2)  18.65 (0.46, 754) | -  0.77  0.02*  0.73  0.03* |
| **Concern for Seizure**, N (%)  0: No  1: Yes  (Deceased/Lost to follow-up, N=4) | 2 (100)  0 (0) | 18 (86)  3 (14) | 2 (17)  10 (83) | 0 (0)  0 (0) | 0.0001** | 1.00  30.4 (2.02, 458) | -  0.0005** |

* P value significant at <.05 (not adjusted using Bonferroni correction). ** P value significant or marginally significant at <.006 (adjusted using Bonferroni correction). ^a^ P value from Fisher exact test; ^b^ P value from ordinal/multinomial logistic regression.

**References**

Boerwinkle VL, Mohanty D, Foldes ST, et al. Correlating Resting-State Functional Magnetic Resonance Imaging Connectivity by Independent Component Analysis-Based Epileptogenic Zones with Intracranial Electroencephalogram Localized Seizure Onset Zones and Surgical Outcomes in Prospective Pediatric Intractable Epilepsy Study. *Brain Connect*. 2017;7(7):424-442. doi:10.1089/brain.2016.0479
